# MRI Reconstruction Using Markov Random Field and Total Variation as Composite Prior

**DOI:** 10.3390/s20113185

**Published:** 2020-06-03

**Authors:** Marko Panić, Dušan Jakovetić, Dejan Vukobratović, Vladimir Crnojević, Aleksandra Pižurica

**Affiliations:** 1 BioSense Institute, University of Novi Sad, 21000 Novi Sad, Serbia; crnojevic@biosense.rs; 2 Faculty of Sciences, University of Novi Sad, 21000 Novi Sad, Serbia; dusan.jakovetic@dmi.uns.ac.rs; 3 Faculty of Technical Sciences, University of Novi Sad, 21000 Novi Sad, Serbia; 4 Department of Telecommunications and Information Processing, Ghent University, 9000 Ghent, Belgium

**Keywords:** magnetic resonance imaging, Markov random field, image reconstruction

## Abstract

Reconstruction of magnetic resonance images (MRI) benefits from incorporating a priori knowledge about statistical dependencies among the representation coefficients. Recent results demonstrate that modeling intraband dependencies with Markov Random Field (MRF) models enable superior reconstructions compared to inter-scale models. In this paper, we develop a novel reconstruction method, which includes a composite prior based on an MRF model and Total Variation (TV). We use an anisotropic MRF model and propose an original data-driven method for the adaptive estimation of its parameters. From a Bayesian perspective, we define a new position-dependent type of regularization and derive a compact reconstruction algorithm with a novel soft-thresholding rule. Experimental results show the effectiveness of this method compared to the state of the art in the field.

## 1. Introduction

Recent years have witnessed huge interest in accelerating Magnetic Resonance Imaging (MRI) acquisition and improving the achievable quality of MRI images based on compressed sensing [1,2,3]. Reconstruction of a magnetic resonance image $x\in C^{N}$ is formulated as the following optimization problem

(1) $$\min_{x}\frac{1}{2}||y-{\mathrm{Ax}||}_{2}^{2}+\tau \phi \left(\mathrm{Bx}\right)$$

where $y\in C^{M}$ are measurements obtained through partially observed Fourier transform $A\in C^{M\times N}$, M≪N [4]. $B\in C^{D\times N}$ denotes analysis operator and ϕ is a regularization function, with the parameter τ>0. ϕ is typically the $\ell_{1}$-norm: $\phi \left(\theta \right)={\left||\theta |\right|}_{1}$ when B=W denotes a wavelet-like transformation of the signal x, e.g, θ=Wx, or the TV pseudo-norm ${\left||x|\right|}_{\mathrm{TV}}$ when B=I is the identity operator. With compound regularization, the problem in (1) becomes

(2) $$\min_{x}\frac{1}{2}||y-{\mathrm{Ax}||}_{2}^{2}+\sum_{i=1}^{m}\tau_{i}\phi_{i}\left(B_{i}x\right).$$

Many reported methods, including [4,5,6,7] combine $\ell_{1}$ and TV regularization: $B_{1}=I$, $B_{2}=W$, $\phi_{1}\left(x\right)={\left||x|\right|}_{\mathrm{TV}}$, $\phi_{2}\left(\mathrm{Wx}\right)={\left||\theta |\right|}_{1}$, which proved to be beneficial over either of these regularization functions alone.

Apart from CS-MRI iterative algorithms for solving the problem in (1), deep learning, which has been successful in solving many computer vision problems, has also been proved beneficial in tackling image reconstruction problems [8,9]. In particular, deep convolutional neural networks (CNNs) are extremely powerful and efficient in learning signal representation [10]. Wang et al. in [11] train a deep CNN from downsampled reconstruction MRI images to learn a fully sampled reconstruction and then use deep learning results either as an initialization or as a regularization term (1). Following the same principle, the authors of [12] trained a reconstruction function from undersampled/full sampled image pairs, utilizing a more complex network structure based on U-net architecture [13] compared to the one used in [11]. In [14], motivated by a linkage between CNNs and Hankel matrix decomposition, the authors propose a fully data-driven deep learning algorithm for *k*-space interpolation of undersampled measurements. A trend of designing complex network architectures for constructing de-aliasing MRI reconstruction function was continued in [15,16] with the involvement of Generative Adversarial Network (GAN) [17] and U-net architectures and in [18,19] where a deep cascaded CNNs and its stochastic variation are used. A GAN based network architecture demonstrated also promising performances in the task of super-resolution (SR) for image enhancement which has great importance in medical image applications [20,21,22,23]. However, besides very good performances, the training procedure of deep CNNs, especially GANs, demands good hyperparameters tuning to achieve training convergence, which without experience is a quite difficult and time-consuming task. Another observation is that most of the results of trained deep CNNs are reported for the brain MRI scans, which means that their usage for the MRI images of another anatomy would possibly require a new training cycle.

Recent research showed that MRI reconstruction benefits from modelling statistically the spatial clustering properties of the important transform coefficients, i.e., modelling the coefficient support. Representatives are wavelet-tree sparsity methods [24,25] and methods that employ MRF priors [26,27,28,29]. A work of Panić et al. [29] demonstrated a superior reconstruction performance of MRF-based approaches over wavelet-tree sparsity methods. The reconstruction algorithm in [29] is an augmented Lagrangian method inspired by [30] and extended with an MRF prior. A variant of this algorithm, also reported in [29] with compound regularization consisting of a MRF-based and TV regularization terms demonstrated significant improvements over several state-of-the-art methods, including [25,31,32]. Although the algorithms in [29] perform well over a wide range of the parameters of the MRF model, on the analysed human brain images, they require parameter tuning when imaging different anatomical structures. We also found that hard-thresholding rule in [29] based on the estimated sparse signal support leads to some small oscillations around the convergence state, which do not affect noticeably the quality of the reconstruction but may impair practical determination of the stopping criterion.

In this paper, we aim to develop a more robust model, with automatic estimation of the parameters and with improved stability. We also extend the isotropic MRF model used in [29] to a more flexible non-isotropic model that can better capture the properties of various anatomic structures and adapt to these automatically through efficient parameter estimation. To solve the resulting optimisation problem, we develop a novel numerical method that involves a Nesterov acceleration step [33,34]. The main technical novelties are in: (i) developing a compressed sensing approach for MRI images with an anisotropic MRF model and deriving automatic estimation of its parameters; (ii) deriving a more stable solver, which replaces hard selection of the coefficients by a soft-thresholding (note that the resulting estimator is derived analytically by introducing the corresponding proximal operators for MRF-based and TV regularization functions); (iii) we extend this approach such that it is applicable not only for the reconstruction of the magnitude images as in [29], but also to complex images and to multi-coil data.

The paper is organized as follows. The formulation of the problem with the proposed regularization function is given in Section 2.1. In Section 2.2 we derive the efficient solver, and in Section 2.3 we develop an efficient method for the estimation of the MRF parameters. Section 2.4 introduces algorithm extensions to reconstruction of complex images from one or more coil measurements. Section 3 presents experimental results and comparison with state-of-the-art methods. In Section 4 we provide our conclusions.

## 2. Methodology

### 2.1. MRF Priors in MRI Reconstruction

Consider a Bayesian approach to recovering jointly representation coefficients θ=Wx and their bi-level significance map s with the maximum a posteriori probability (MAP) criterion: P(θ,s|y)∝p(y|θ)p(θ|s)P(s). Here, $s\in {\left\{-1,1\right\}}^{D}$ are the latent variables formed in a lattice that encode the significance of the representation coefficients: $s_{i}=+1$ on those positions *i* in lattice where $\theta_{i}$ is significant, and $s_{i}=-1$ conversely. P(s) is the prior probability of s while p(y|θ) and p(θ|s) are conditional probability density functions (p.d.f.). Equivalent formulations of the graphical model were presented, e.g., in [27,28,29] and a similar one in [26,35] for the signal sparse in image domain. Using the negative log transformation of P(θ,s|y), the MAP criterion translates into the joint minimization with respect to (w.r.t.) θ and s:

(3) $$\min_{\theta ,s}-\left(\log p(y|\theta )+\log p(\theta |s)+\log P(s)\right).$$

We make a connection between (3) and formulations of type (1) by reordering the terms as follows:

(4) $$\min_{\theta }\left\{\underset{f(W^{H}\theta )}{\underset{︸}{-\log p(y|\theta )}}+\underset{\psi_{\mathrm{MRF}}\left(\mathrm{Wx}\right)}{\underset{︸}{\min_{s}-\left(\log p(\theta |s)+\log P(s)\right)}}\right\}.$$

**Figure 1:**
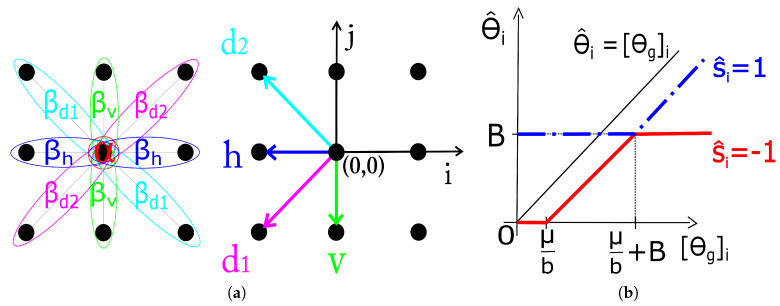
(**a**) Single and pair-site cliques for the second-order neighborhood with parameters and directions used in calculation of $R_{t}$. (**b**) Soft-thresholding rules for image coefficients ${\left[\theta_{g}\right]}_{i}$ based on the estimated ${\hat{s}}_{i}=\left\{1,-1\right\}$.

Assuming that measurements y are corrupted by independent identically distributed (i.i.d.) Gaussian noise, we have that p(y|θ) = $N(y|{\mathrm{AP}}^{H}\theta ,I)$. It then follows that the first term $f(W^{H}\theta )$ in (4) using a relation $x=W^{H}\theta$ becomes equal to data fidelity part $\frac{1}{2}||y-{\mathrm{Ax}||}_{2}^{2}$ in (1). $\psi_{\mathrm{MRF}}\left(\mathrm{Wx}\right)$ is the new type of regularization function, which is taking the role of the second term τϕ(Bx) term from (1). Assuming conditional independence $p\left(\theta |s\right)=\prod_{i}p\left(\theta_{i}|s_{i}\right)$ and a Laplacian prior for image coefficients $p(\theta_{i})$ we define accordingly conditionals $p(\theta_{i}|s_{i})$ as follows: $p\left(\theta_{i}|s_{i}=1\right)\propto \frac{1}{2b}e^{-\frac{|\theta_{i}|}{b}}$ for $|\theta_{i}|\geq B$ and zero otherwise while $p(\theta_{i}|s_{i}=-1)$ is defined oppositely with respect to *B*. The threshold *B* is related to the noise level and estimated from the highest-frequency subband coefficients; the scale parameter *b* is adaptively estimated per subband [29]. We choose the Gibbs distribution (MRF prior) for the θ’s support model $P\left(s\right)=\frac{1}{Z}e^{-H(s)/T}$, where the normalizing constant $Z=\sum_{s}e^{-H(s)/T}$ represents the partition function, and the temperature *T* controls the peaking in the probability density [36]. We employ a common model with pair-site cliques. However, in contrast to our previous work [27,28,29], we allow for different interaction coefficients for cliques of different orientations, which models better the actual subband statistics. The energy function of this anisotropic MRF is

(5) $$H\left(s\right)=\sum_{i}\alpha s_{i}+\sum_{\langle i,j\rangle \in C}\beta_{o}s_{i}s_{j}$$

where α expresses the a priori preference for labels of one type over the other and $\beta_{o}$ the interaction strength for cliques with orientation o∈{h,v,d1,d2} shown in Figure 1. The estimation of the MRF parameters ($\beta_{o},\alpha$) is treated in Section 2.3.

Involving the composition of the MRF-based regularization function $\psi_{\mathrm{MRF}}\left(\mathrm{Wx}\right)$ and TV norm $\phi_{\mathrm{TV}}\left(x\right)={\left||x|\right|}_{\mathrm{TV}}$, defined as ${∥x∥}_{\mathrm{TV}}=\sum_{i}\sum_{j}\sqrt{|x_{i+1,j}-x_{i,j}{|}^{2}+{\left|x_{i,j+1}-x_{i,j}\right|}^{2}}$, we arrive at the proposed optimization problem formulation:

(6) $$\min_{x}\left\{f\left(x\right)+\psi_{\mathrm{MRF}}\left(\mathrm{Wx}\right)+\phi_{\mathrm{TV}}\left(x\right).\right\}$$

The optimization problem (6) is non-convex in general due to the presence of the non-convex $\psi_{\mathrm{MRF}}$ regularization function and is very hard to globally solve exactly. In the following section we propose a computationally efficient method to suboptimally solve (6).

### 2.2. Proposed Algorithm

To solve the problem (6) we adopt the idea of fast composite splitting from [37] and extend this algorithm to deal with the MRF-based regularization. The key difference is that our model involves $\psi_{\mathrm{MRF}}$ instead of $\ell_{1}$-norm in [37]. Hence, we have to solve the proximal map $\hat{\theta }={\mathrm{prox}}_{\mu }\left(\psi_{\mathrm{MRF}}\right)\left({\mathrm{Wx}}_{g}\right)$:

(7) $$\underset{\theta }{\mathrm{argmin}}\left(\psi_{\mathrm{MRF}}\left(\theta \right)+\frac{1}{2\mu }{∥\theta -{\mathrm{Wx}}_{g}∥}_{2}^{2}\right).$$

Note that, in notation ${\mathrm{prox}}_{\mu }\left(\psi_{\mathrm{MRF}}\right)\left({\mathrm{Wx}}_{g}\right)$, $\psi_{\mathrm{MRF}}$ is the function for which the proximal map is calculated, and $\theta_{g}={\mathrm{Wx}}_{g}$ is the argument at which it is evaluated. Since the evaluation of this proximal map is very hard, we adopt a suboptimal, block-coordinate approach explained in the following.

The key novel ingredient of the proposed algorithm is a computationally efficient approximation of the proximal map in (7) for a fixed $x_{g}$. From (4), it is clear that the minimization in (7) needs to be carried out jointly w.r.t. θ and s in order to evaluate (7) exactly. We adopt a suboptimal yet computationally efficient approach where we first minimize the second term in (4) w.r.t. $s\in {\left\{-1,1\right\}}^{D}$ for a fixed θ; then we fix the obtained $\hat{s}$ and minimize the objective in (7) w.r.t. θ. For the former minimization step, we adopt notation $\hat{s}=\mathrm{MAP}-\mathrm{support}\left\{\theta \right\}$. The step is done via the Metropolis sampler procedure using a “warm-start” initial s as in [28,29]. The latter minimization (w.r.t. θ for a fixed $\hat{s}$), as shown here, can be done in closed-form and leads to a novel soft-thresholding operation. This is accomplished by solving the following minimization problem

(8) $$\hat{\theta }=\underset{\theta }{\mathrm{argmin}}\underset{\psi_{\hat{s}}\left(\theta \right)}{\underset{︸}{\sum_{i}-\log p\left(\theta_{i}|{\hat{s}}_{i}\right)}}+\frac{1}{2\mu }{\left(\theta_{i}-{\left[\theta_{g}\right]}_{i}\right)}^{2}.$$

Equation (8) is derived from (7) using a simple algebraic manipulation and omitting the terms that do not depend on θ. With the analytic form of $p_{\Theta_{i}|S_{i}}\left(\theta_{i}|s_{i}\right)$ for ${\hat{s}}_{i}=\left\{-1,1\right\}$ (see Section 2.1), the closed form solution for each single component of $\hat{s}$ (it turns out that the solution decouples component-wise) is derived in (9) (index *i* in the equation is omitted for notation simplicity). The solution (9) is illustrated in Figure 1 on range [0,∞) due to its (odd) symmetry about the origin.

(9) $$\begin{array}{cc} {\hat{\theta }}_{\hat{s}=1} & =\left\{\begin{array}{cc} B\cdot \mathrm{sgn}\left(\theta_{g}\right), & |\theta_{g}|\leq \frac{\mu }{b}+B\\ \theta_{g}-\frac{\mu }{b}\cdot \mathrm{sgn}\left(\theta_{g}\right), & |\theta_{g}|>\frac{\mu }{b}+B \end{array}\right.\\ {\hat{\theta }}_{\hat{s}=-1} & =\left\{\begin{array}{cc} 0, & |\theta_{g}|\leq \frac{\mu }{b}\\ \theta_{g}-\frac{\mu }{b}\cdot \mathrm{sgn}\left(\theta_{g}\right), & \frac{\mu }{b}<\left|\theta_{g}\right|\leq \frac{\mu }{b}+B\\ B\cdot \mathrm{sgn}\left(\theta_{g}\right), & |\theta_{g}|>\frac{\mu }{b}+B \end{array}\right. \end{array}$$

The described block-coordinate cyclic procedure should in principle proceed in several iterative rounds. As we demonstrate here numerically, it is sufficient to perform a single cycle. Once the approximation of the proximal map in (7) is available, we incorporate it in a fast-splitting framework akin to the one in [37]. That is, we first carry out a gradient-like step on x w.r.t. data fidelity term. Then, we perform in parallel the proximal maps that correspond to the two regularization functions in (6). The two outputs of the proximal mappings are then simply averaged and fed into a Nesterov-acceleration-like step. The motivation for the parallel-proximal-then-average approach is computational efficiency, as carrying out a joint proximal map (even approximate) w.r.t. $\psi_{\mathrm{MRF}}$ and $\phi_{\mathrm{TV}}$ would be very hard. The motivation for the Nesterov-acceleration step is to further speed-up the method.

The overall method is presented in Algorithm 1. Recall the definition of $\psi_{\hat{s}}$ from (8). Note that steps 3-4 are the single block-coordinate cycle to approximate the operation in (7). The parameters $\tau_{1},\tau_{2},\mu$ for simplicity are all set to 1, although these are not the optimal values, unless it is otherwise stated. We refer to the proposed method as Fast Composite Lattice and TV regularization (FCLaTV). A version without acceleration CLaTV can also be used, and it is obtained after omitting steps 7 and 8 from FCLaTV and replacing $r^{\left\{k\right\}}$ in the step 1 with $x^{\left\{k\right\}}$. Extensive numerical studies demonstrate that Algorithm 1 always converges. The rigorous convergence analysis is left for future work.

**Algorithm 1** FCLaTV
**Require:**
$k=1,\mu ,\tau_{1},\tau_{2},t^{\left\{1\right\}}=1,y,r^{\left\{1\right\}}=x^{\left\{0\right\}}$
1:
**repeat**
2:    $x_{g}=r^{\left\{k\right\}}-\mu A^{H}\left(Ar^{\left\{k\right\}}-y\right)$3:    $\left[\psi_{\hat{s}},\hat{s}\right]=\mathrm{MAP}-\mathrm{support}\left\{{\mathrm{Wx}}_{g}\right\}$4:    $x_{1}=W^{H}\left({\mathrm{prox}}_{\mu }\left(\tau_{1}\psi_{\hat{s}}\right)\left({\mathrm{Wx}}_{g}\right)\right)$5:    $x_{2}={\mathrm{prox}}_{\mu }\left(\tau_{2}\phi_{\mathrm{TV}}\right)\left(x_{g}\right)$6:    $x^{\left\{k\right\}}=\left(x_{1}+x_{2}\right)/2$7:    $t^{\left\{k+1\right\}}=(1+\sqrt{1+4{\left(t^{\left\{k\right\}}\right)}^{2}})/2$8:    $r^{\left\{k+1\right\}}=x^{\left\{k\right\}}+\frac{t^{\left\{k\right\}}-1}{t^{\left\{k+1\right\}}}\left(x^{\left\{k\right\}}-x^{\left\{k-1\right\}}\right)$9:    k=k+110:**until** some stopping criterion is satisfied11:
**return** $x=x^{\left\{k\right\}}$



### 2.3. Parameter Estimation for the Anisotropic MRF Prior

Here we propose a data-driven approach for specifying the parameters $\left\{\alpha ,\beta_{h},\beta_{v},\beta_{d1},\beta_{d2}\right\}$ of the MRF model. The core idea is to relate the parameters of the prior P(s) for the support of θ to some measurable characteristics of the observed θ. The representation coefficients θ are re-estimated in each iteration of Algorithm 1 and so are the MRF parameters.

The four interaction coefficients $\left\{\beta_{h},\beta_{v},\beta_{d1},\beta_{d2}\right\}$ represent the clustering strength of the coefficient labels s in the corresponding four directions for the observed subband. We reason that the clustering strength in a particular direction should be proportional to the correlation among the corresponding representation coefficients θ in that direction. Therefore, we base the estimation of interaction coefficients on magnitude of representation coefficients θ. In order to reduce the effect the noise present in θ on estimation of the interaction coefficients, we used squared magnitude of θ and account only for those that were marked as significant by the initial s prior to its MAP estimation $\hat{s}$. In what follows, $\theta^{S}$ denotes a subband in which all the coefficients that were selected as significant by s are left unchanged ($\theta_{i}^{S}=\theta_{i}$ if $s_{i}=1$) while others are set to zero ($\theta_{i}^{S}=0$ if $s_{i}=-1$). To express two-dimensional (2D) correlation, we need to revert to 2D spatial indices (k,l). Let $r_{\left(i,j\right)}$ denote the correlation coefficient for squared $\theta^{S}$ corresponding to the spatial shift (i,j):

(10) $$r_{\left(i,j\right)}=\sum_{k}\sum_{l}{\left(\theta^{S}\left(k,l\right)\theta^{S}\left(k+i,l+j\right)\right)}^{2}$$

We take four correlation coefficients corresponding to the smallest possible spatial shifts in the corresponding directions, indicated by arrows in Figure 1. These are: $r_{\left(-1,-1\right)}$, $r_{\left(-1,0\right)}$, $r_{\left(-1,1\right)}$ and $r_{\left(0,-1\right)}$. Note that by symmetry $r_{\left(-1,-1\right)}=r_{\left(1,1\right)}$, $r_{\left(-1,0\right)}=r_{\left(1,0\right)}$, $r_{\left(-1,1\right)}=r_{\left(1,-1\right)}$ and $r_{\left(0,-1\right)}=r_{\left(0,1\right)}$. The mapping t=3i+j+4 translates these index pairs into a single index t∈{0,1,2,3}. With $R_{t}=r_{\left(i,j\right)}$ we have that $R_{0}=r_{\left(-1,-1\right)}$ is the correlation coefficient in the 45${}^{\circ }$ ($d_{1}$-direction), $R_{1}=r_{\left(-1,0\right)}$ the horizontal, $R_{2}=r_{\left(-1,1\right)}$ the 135${}^{\circ }$ ($d_{2}$-direction) and $R_{3}=r_{\left(0,-1\right)}$ in the vertical direction. Now we can specify the four interaction coefficients as normalized correlation coefficients of $\theta^{S}$:

(11) $$\begin{array}{cc} \beta_{d1} & =\frac{R_{0}}{{∥R∥}_{2}},\;\beta_{h}=\frac{R_{1}}{{∥R∥}_{2}}\\ \beta_{d2} & =\frac{R_{2}}{{∥R∥}_{2}},\;\beta_{v}=\frac{R_{3}}{{∥R∥}_{2}} \end{array}$$

The normalization by $\ell_{2}$-norm here is optional (as only the relative values of the MRF parameters with respect to each other actually matter) but we find it convenient in practice to have these parameters in the range [−1,1] as it is now guaranteed. We also tested $\ell_{1}$ and $\ell_{\infty }$ for the purpose of this normalization, but $\ell_{2}$ led to best performances in our experiments.

It still remains to specify the parameter α, which represents a priori preference for one type of labels (−1 or +1) over the other. With α=0 both labels are a priori equally likely and as α increases in magnitude the more preference goes to one of these labels. For α>0, the labels −1 will be favoured, which means that significant coefficients (labelled by +1) will be sparse. We specify α as the mean energy of the coefficients in $\theta^{S}$ relative to the energy of the largest coefficient in that subband:

(12) $$\alpha =\frac{1}{N}\frac{{∥\theta^{S}∥}_{2}^{2}}{{\left({∥\theta^{S}∥}_{\max}\right)}^{2}}$$

Note that we have omitted the iteration indices for compactness. In fact, we have sequences $s^{\left(k\right)}$, $\theta^{S(k)}$ and $\alpha^{\left(k\right)}$, $\beta_{h}^{\left(k\right)}$, $\beta_{v}^{\left(k\right)}$, $\beta_{d1}^{\left(k\right)}$, $\beta_{d2}^{\left(k\right)}$ that get improved through iterations *k*.

### 2.4. Complex Image Reconstruction and Multi-Coil Reconstruction

The proposed approach is developed for the reconstruction of MR image magnitude. Here we extend it for the reconstruction of complex images (magnitude and phase) and the reconstruction of magnitude images from multi-coil measurements. In cases where it is of interest to recover the image phase together with the magnitude, steps 4 and 5 in Algorithm 1 are repeated twice: first for the regularization of the real part and then, equivalently, for the regularization of the imaginary part of a complex image x. This approach showed promising performances as it will be seen in the following section. A multi-coil image reconstruction demands knowledge of sensitivity profiles for each coil and therefore different construction of the operator A during the reconstruction procedure. If we denote with $C_{i}$ the sensitivity profile for coil *i* then undersampled measurements from the same coil are $y_{i}={\mathrm{AC}}_{i}x$. Let us collect all available measurements from $N_{c}$ coils in one vector $y={\left[y_{1}^{T},y_{2}^{T}...y_{N_{c}}^{T}\right]}^{T}$ and create an augmented vectorized image $x_{a}=Tx$ with the usage of matrix T which is formed by stacking the identity matrix $I_{N\times N}$$N_{c}$ times row-wise. Then the acquisition process is defined through the following equation

(13) $$y=A_{B}C_{B}x_{a}$$

where $A_{B}$ consists of repeated A along the diagonal while $C_{B}$ is formed by stacking coil sensitivity maps $C_{i}$ along diagonal. This way a multi-coil reconstruction problem can be solved using the single-coil reconstruction method developed in this paper. This generalization in the definition of the measurement operator $M=A_{B}C_{B}$ is easily introduced in step 2 in Algorithm 1 instead of A corresponding to the single-coil reconstruction scenario. The computational complexity is marginally increased and doesn’t require additional code optimization.

## 3. Results

**Figure 2:**
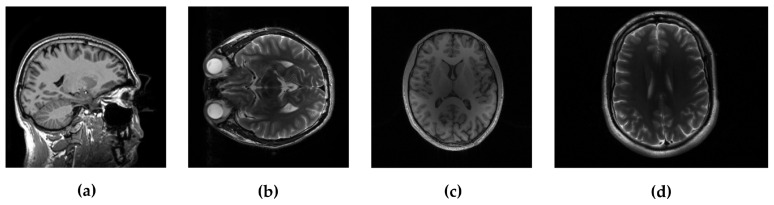
Test images: (**a**) *sagittal*, (**b**) *axial-1* and (**c**) *axial-2*, all three 256×256, and (**d**) *axial-3* with resolution 256×340.

In the experimental evaluation we used different MRI images, starting from high-resolution MRI images, shown in Figure 2, and using real data acquired in *k*-space. For the following experiments, we used simulated sampling trajectories on a Cartesian grid with different sampling rates, except for the last experiment, when the measurements are undersampled with the non-Cartesian radial sampling trajectory used in a real scan. First, we consider reconstruction of MR image magnitude for different sampling rate (SR) obtained using various sampling trajectories. For this we utilize dataset of 248 T1 MRI brain slices acquired on a Cartesian grid at Ghent University hospital (one *sagittal* slice is presented in Figure 2). Then we focus on the reconstruction of complex MR images from single and multi-coil undersampled measurements. Complex T2-weighted brain images *axial-1, axial-3* from [31] and [38] respectively (their magnitudes are presented in Figure 2) are used in experiments for the reconstruction of single-coil complex images while the T1-weighted brain image *axial-2* [38] is used in the multi-coil reconstruction experiment. We also present the reconstruction results on real radially acquired measurements in *k*-space on non-Cartesian grid. These data, consisting of the acquisitions of a pomelo fruit were supplied by the Bioimaging Lab in Antwerp. In our experiments for sparse signal representation we used the non-decimated wavelet transform with 3 scales. For comparison, we report the results of LaSAL and LaSAL2 from [29], FCSA [5], FCSANL [39] and WaTMRI [25] with the original implementations. All these methods, except LaSAL, employ a compound regularization. The reconstruction results for complex images from single-coil and multi-coil measurements, are compared with the corresponding results of pFISTA [31] and P-LORAKS [38,40] methods.

For quantitative comparison between the reconstructed and the reference MR image we adopt Peak Signal to Noise Ratio (PSNR) and Structural Similarity Index (SSIM). PSNR is defined as:

(14) $$PSNR\left(\hat{x},x_{r}\right)=10\log_{10}\left(\frac{MAX_{MR}^{2}}{MSE(\hat{x},x_{r})}\right)$$

where $MAX_{MR}^{2}$ denotes squared maximum possible pixel value in the magnitude of MR image and MSE is the mean squared error between magnitudes of reconstructed $\hat{x}$ and reference image $x_{r}$. For the calculation of SSIM index we used the simplified equation form:

(15) $$SSIM\left(\hat{x},x_{r}\right)=\frac{\left(2\mu_{\hat{x}}\mu_{x_{r}}+C_{1}\right)\left(2\sigma_{\hat{x}x_{r}}+C_{2}\right)}{\left(\mu_{\hat{x}}^{2}+\mu_{x_{r}}^{2}+C_{1}\right)\left(\sigma_{\hat{x}}^{2}+\sigma_{x_{r}}^{2}+C_{2}\right)}$$

where $\mu_{\hat{x}},\mu_{x_{r}},\sigma_{\hat{x}},\sigma_{x_{r}},\sigma_{\hat{x}x_{r}}$ are the local means, standard deviations, and cross-covariance for images $\hat{x}$, $x_{r}$ and $C_{1}={\left(0.01*MAX_{MR}\right)}^{2}$ and $C_{2}={\left(0.03*MAX_{MR}\right)}^{2}$ are regularization constants to avoid instability in index calculation. Local statistics is calculated using isotropic Gaussian function with radius 1.5. Through experiments pixel values are stored in double-precision floating-point format with dynamic range of [0,255] unless otherwise stated.

### 3.1. Data Sets Acquired on Cartesian Grid

**Figure 3:**
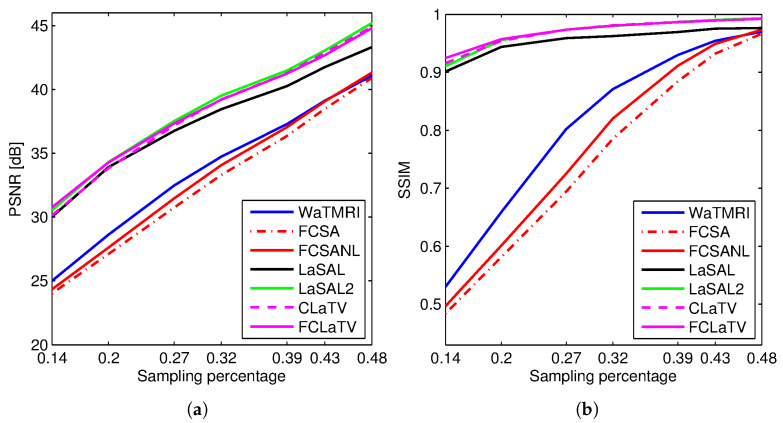
(**a**) PSNR and (**b**) SSIM for the reconstructions of the test image *sagittal* at different sampling rates.

Figure 3 shows the PSNR and SSIM for the reconstructed *sagittal* MR image from radially undersampled measurements with sampling rate (SR) ranging from 14% to 48%. The MRF-based methods LaSAL, LaSAL2, CLaTV and FCLaTV achieve a consistent and significant improvement in PSNR (at some sampling rates more than 4 dB) compared to WaTMRI, FCSA and FCSANL. The proposed methods CLaTV and FCLaTV outperform LaSAL and yield only slightly lower reconstruction PSNR and equally good SSIM as the best reference method LaSAL2. These results are achieved with automatic estimation of MRF parameters and without tuning the regularization parameters (the μ,$\tau_{1}$ and $\tau_{2}$ parameters are all set to 1).

**Figure 4:**
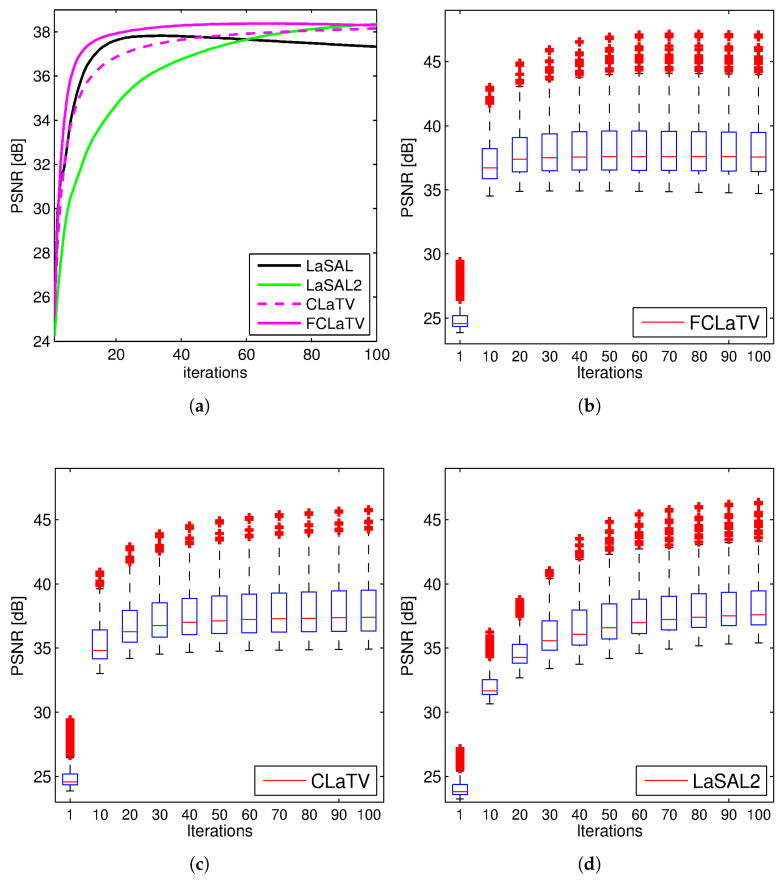
PSNR values obtained from 248 MRI brain slices from the first data set, with radial sampling (SR 25%). Mean PSNR (**a**) and the PSNR distribution for FCLaTV (**b**), CLaTV (**c**), LaSAL2 (**d**). The result is presented as a box plot: the edges of the each box represents 25th and 75th percentile while the central mark (red line) in the box is median. The whiskers extend to the most extreme PSNR values which are not considered outliers while outliers are plotted separately with red crosses.

Performances of the proposed methods are further tested through reconstruction of all 248 T1 MRI slices from data set. Results are shown in Figure 4 where besides mean PSNR values through iterations we also provide distribution of PSNR values for CLaTV, FCLaTV and LaSAL2 methods. On average, FCLaTV reached the peak performance much before CLaTV and LaSAL2. The LaSAL2 achieved its highest PSNR in average after 80 iterations which is 2 times more after FCLaTV. CLaTV, FCLaTV and LaSAL2 reached the same maximal median PSNR value of 37.5 dB through all iterations. The proposed methods need less iterations than LaSAL2 to reach this value which is presented in Figure 4 with the shift of PSNR distribution towards higher values in the first 20 iterations.

**Figure 5:**
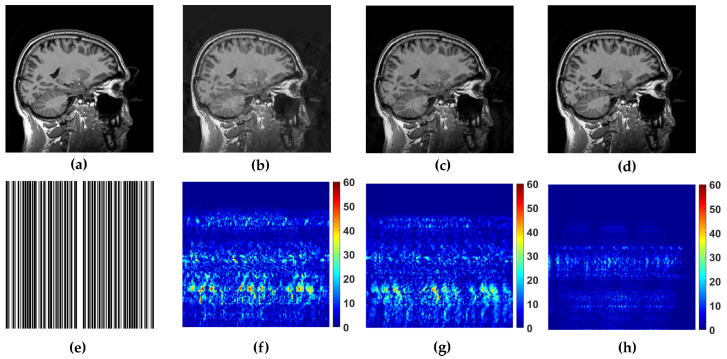
Reference image (**a**) *sagittal* and its reconstruction with the folowing methods (**b**) WaTMRI (PSNR = 28.70 dB, SSIM = 0.81), (**c**) LaSAL2 (PSNR = 29.77 dB, SSIM = 0.84) and (**d**) FCSLa (PSNR = 36.49 dB, SSIM = 0.97) using 45% of measurements sampled with trajectory shown in (**e**). The corresponding reconstruction error of the used methods are shown in (**f**–**h**).

WaTMRI, LaSAL2 and FCLaTV are further tested in reconstruction from measurements undersampled with straight vertical lines in *k*-space which is usual sampling trajectory in real scanners. From the results of this experiment, shown in Figure 5, the FCLaTV method outperforms WaTMRI and LaSAL2 visually and in terms of PSNR and SSIM measure.

**Figure 6:**
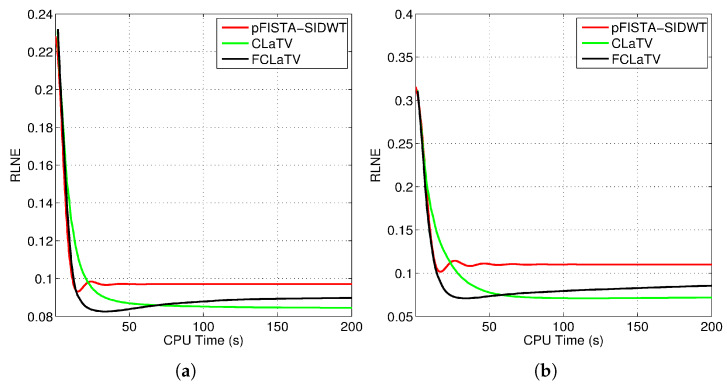
Obtained RLNE for the reconstructed T2-weighted brain image *axial-1* from (**a**) radially and (**b**) randomly undersampled measurements with the same SR of 30%.

We tested the proposed methods in the reconstruction of a complex T2-weighted MR image *axial-1* slice, the magnitude of which is shown in Figure 2. For the performance measure we use relative $\ell_{2}$ norm error (RLNE), defined as

(16) $$e\left(\hat{x}\right)=\frac{{∥\hat{x}-x_{r}∥}_{2}}{{∥x_{r}∥}_{2}}$$

where $x_{r}$ is the reference image recovered from all measurements while $\hat{x}$ denotes the estimated (reconstructed) image from undersampled measurements. Algorithm steps 4 and 5, which refer to proximal operators are simultaneously applied on real and imaginary parts of the temporary reconstructed image $x_{g}$ obtained from step 2. We found that this adaptation of algorithm for handling reconstruction of complex images achieves the best performances. For the reference methods we use the pFISTA method from [31] with the Shift Invariant Discrete Wavelet Transform (SIDWT) as a tight frame for sparse signal representation. Results are shown in Figure 6 with respect to amount of CPU (Intel(R) Core(TM) i7-4700MQ CPU @ 2.4GHz, 8GB of RAM memory) time needed for reconstruction. For the both trajectories, CLaTV and FCLaTV outperform pFISTA-SIDWT in terms of RLNE measure. FCLaTV in less than 50s reaches the lowest RLNE for both trajectories, 0.0826 for radial and 0.0709 for random, while CLaTV needs around 70s to reach RLNE of 0.0846 for radial and 0.0711 for random trajectory.

A comparison of FCLaTV with the P-LORAKS [40] method is conducted by reconstructing the single channel T2-weighted complex brain image *axial-3* from 50% of measurements sampled by random and uniform trajectory. The results are presented in Table 1 where for both sampling strategies FCLaTV achieved lower RLNE value than the reference P-LORAKS method.

**Figure 7:**
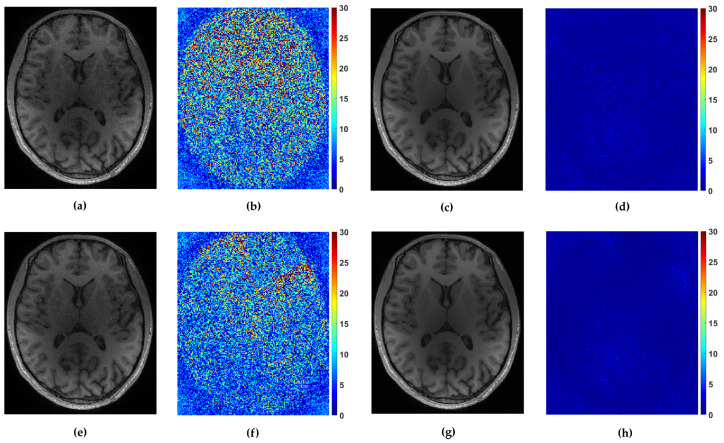
Reconstruction of *axial-2* from randomly and uniformly sampled measurements from 4 coils. (**a**,**c**) Reconstructed images with P-LORAKS and FCLaTV method respectively from randomly sampled measurements with their (**b**,**d**) corresponding errors. (**e**,**g**) Reconstructed images with P-LORAKS and FCLaTV method respectively from uniformly sampled measurements with their (**f**,**h**) corresponding errors.

For the multi-coil reconstruction scenario, we used a T1 weighted brain image *axial-2* from [38], shown in Figure 2, and measurements gathered from 4 coils with known given sensitivity profiles. We incorporate random and uniform sampling trajectories with sampling rate of 14% per each coil. For both trajectories, the proposed FCLaTV achieved lower RLNE values reported in Table 1. Figure 7 shows visual comparison of reconstructions between FCLaTV and P-LORAKS methods.

### 3.2. Data Sets Acquired on Non-Cartesian Grid

**Figure 8:**
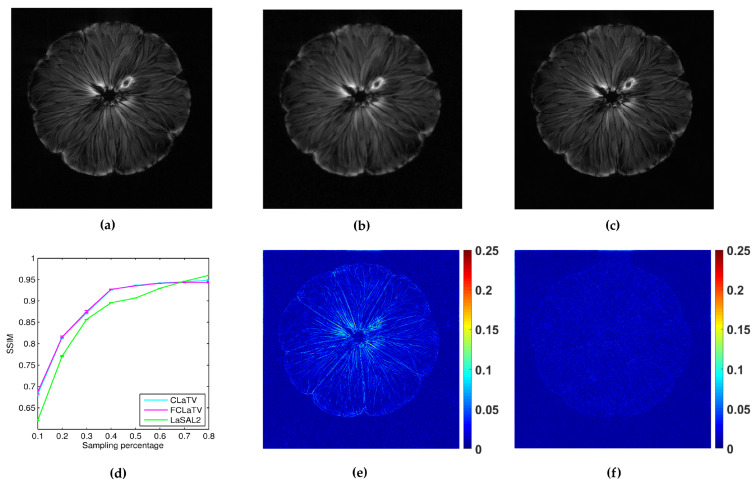
Reconstruction of pomelo fruit from radially sampled measurements: (**a**) reference image obtained by conjugate gradient method using all measurements, (**b**,**c**) pomelo reconstructions using LaSAL2 and FCLaTV methods respectively from 40% of measurements, (**d**) average SSIM for different sampling rate, (**e**,**f**) corresponding reconstructions errors using LaSAL2 and FCLaTV methods respectively.

In the following we present the reconstruction of a *pomelo*, acquired with radial sampling in the *k*-space. The data consist of 1608 radial lines, each with 1024 samples. We form undersampled versions by leaving out some of the radial lines. In particular, we aim to implement undersampling based on the golden ratio profile spacing [41], which guarantees a nearly uniform coverage of the space for an arbitrary number of the remaining radial lines. Starting from an arbitrary selected radial line, each next line is chosen by skipping an azimuthal gap of $111.246^{\circ }$ until we reach desired sampling rate. In practice we cannot always achieve this gap precisely (since we have a finite, although large, number of lines to start with). Therefore we choose the nearest available radial line relative to the position obtained after moving. Since we deal here with non-uniformly sampled *k*-space data, we need to employ the non-uniform FFT procedures [41], which are commonly used in MRI reconstruction and readily available. For the reference method we used LaSAL2 [29]. During the reconstruction we add different amount of Gaussian noise on undersampled measurements with zero mean and the following standard deviations: 0.01, 0.02, 0.03, 0.04 and 0.05. For each amount we perform 10 experiments of reconstruction with different noise realizations. From the obtained average performances of reconstruction in terms of SSIM for different standard deviation of noise, we calculate average across all suggested standard deviations and present results in the form of mean line with standard deviation bars in Figure 8. We empirically set $\tau_{2}$ to 140 and 130 for CLaTV and FCLaTV respectively due to the different dynamic range of reference image [0,1]. Regularization parameters for LaSAL2 method are taken from [29]. As expected, standard deviation of performances reduce as the sampling rate increase and the mean SSIM becomes greater for all methods. At relatively small sampling rates, up to 60% CLaTV and FCLaTV outperforms LaSAL2 which is presented in visual comparison shown in the same Figure 8. CLaTV and FCLaTV much better recover structure in the image than LaSAL2 which is demonstrated visually in the images of reconstruction error.

## 4. Conclusions

We considered the problem of MRI image reconstruction and formulated it as an optimization problem with a quadratic least-squares type fidelity term and a composite regularization—the sum of a total variation and a MRF regularizer. We propose a novel efficient iterative method to solve the problem that involves proximal maps and Nesterov-like acceleration. The major technical novelty is in a computationally efficient method for approximating the MRF-regularizer proximal map; the method exhibits a novel soft-thresholding rule that is different from standard $\ell_{1}$-norm-induced soft-thresholding. Another major novelty is an efficient method for estimating the MRF model parameters on the fly, during the algorithm iterations. Significant improvements in the reconstruction performance are achieved compared to the related methods. Further research direction will include an extension of evaluation metrics with perceptual quality based and semantic-based metrics [42] and involving more undersampling trajectories from the real clinical scans.

## Figures and Tables

**Table 1:** P-LORAKS [40] and FCLaTV comparison on a T1-weighted *axial-2* and T2-weighted *axial-3* image using random and uniform sampling trajectories.

	*axial-2*		*axial-3*	
	**Random**	**Uniform**	**Random**	**Uniform**
P-LORAKS	0.06	0.07	0.083	0.097
FCLaTV	0.015	0.013	0.078	0.080
